# Nanoparticle-mediated diagnosis, treatment, and prevention of breast cancer

**DOI:** 10.1039/d3na00965c

**Published:** 2024-05-23

**Authors:** Lipsa Leena Panigrahi, Pallavi Samal, Sameer Ranjan Sahoo, Banishree Sahoo, Arun Kumar Pradhan, Sailendra Mahanta, Sandip Kumar Rath, Manoranjan Arakha

**Affiliations:** a Center For Biotechnology, Siksha O Anusandhan University Bhubaneswar Odisha 751003 India marakha@soa.ac.in manoranjan.arakha@gmail.com; b School of Pharmacy, The Assam Kaziranga University Koraikhowa, NH-37 Jorhat Assam 785 006 India; c Department of Radiation Oncology, Winship Cancer Institute, Emory University School of Medicine Atlanta Georgia USA

## Abstract

By virtue of their advanced physicochemical properties, nanoparticles have attracted significant attention from researchers for application in diverse fields of medical science. Breast cancer, presenting a high risk of morbidity and mortality, frequently occurs in women and is considered a malignant tumor. Globally, breast cancer is considered the second leading cause of death. Accordingly, its poor prognosis, invasive metastasis, and relapse have motivated oncologists and nano-medical researchers to develop highly potent nanotherapies to cure this deadly disease. In this case, nanoparticles have emerged as responsive platforms for breast cancer management, providing new approaches to improve the diagnostic accuracy, deliver targeted therapies, and limit the progression of this disease. Recently, smart nano-carriers encapsulating drugs, ligands, and tracking probes have been developed for the specific therapy of breast cancers. Further, efforts have been devoted to developing various nano-systems with minimal toxicity. The aim of this review is to present a background on novel nanotheranostic methods that can be employed to diagnose and treat breast cancers and encourage readers to focus on the development of novel nanomedicine for breast cancers and other deadly diseases. In this context, we discuss different methods for the diagnosis, treatment, and prevention of breast cancers using different metal and metal oxide nanoparticles.

## Abstract 2

Nanoscale materials offer prospects of targeted treatment, precise delivery of therapeutic agents, stimuli-responsive mechanisms, and reduced toxicity in breast cancer patients.

## Graphical abstract

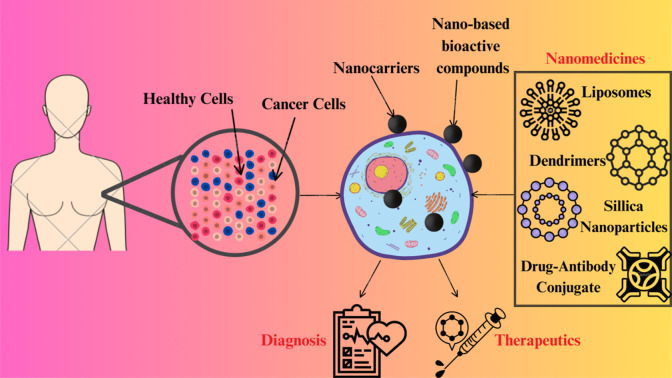

## 1. Introduction

**Fig. 1:**
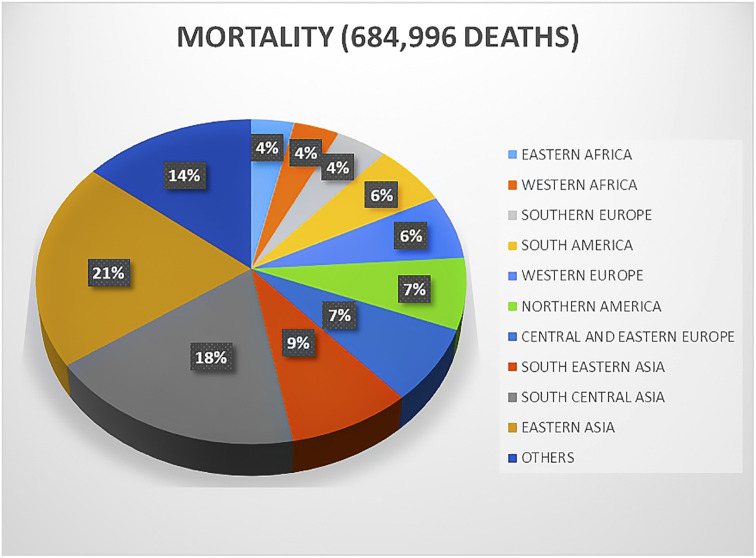
Distribution of breast cancer deaths worldwide.

Breast cancer has rapidly emerged as a crucial health issue faced by women in the past seven decades.^1^ Different specialties including surgery, gynecology, pathology, radiology, hematology, oncology, nuclear medicine, and radiation oncology are engaged in breast cancer therapies. Regarding new cases, about 268 000 new cancer afflictions were diagnosed according to a report by the American Cancer Research Society, which also noted an increasing rate of 1.5% in incidences.^1^ The global statistics according to a 2020 report illustrated that breast cancer is quite alarming, totaling an estimated 19 million new cases and 10 million deaths annually.^2^ Nearly 11 million breast cancer deaths are expected by 2030. Developed countries such as Australia, New Zealand, Western and Northern Europe, and North America are at a higher risk (55 cases per 100 000 individuals) of developing breast cancer compared to developing countries such as Central America, Eastern and Middle Africa, and South-Central Asia (29.7 cases per 100 000 individuals). Furthermore, developed countries have shown 17% higher mortality rates than developing countries.^2^ However, in recent years, a noticeable surge in breast cancer incidences has been reported in developing countries (Fig. 1). Widely proclaimed as the most prevalent ailment in women, breast cancer is widely diagnosed in adolescent and middle-aged women. Age represents the prime risk factor for breast cancer, followed by an overdose of estrogen, early menarche, high intake of progestin-enhancing drugs, late menopause, physical inactivity, obesity, and avoidance of lactation, clearly indicating that breast cancer is the orthodox cancerous complication of resourceful nations and countries. Surgery-based cancer therapy started around 120 years ago by the assessment of mastectomy and its scientific description by Halstead.^3,4^ The outcome was a groundbreaking amalgamation of surgery and science as well as a proactive indigenous solution to a recurring disease, cancer. At present, surgery, radiotherapy, and chemotherapy are mostly employed to treat breast cancer.^3,4^

Breast cancer is an individual catastrophe, where the ease of first-class care, in-time diagnosis, efficient surgical and medical procedures can stabilize or destabilize a patient. Simultaneously, it presents an overall burden given that screening programs and diagnosis are expensive and inefficient to procure and maintain. Proper surgical treatments demand modern treatment and functionalities involving local radiation, which are most of the time missing or unavailable in the healthcare system in underdeveloped countries.^4,5^ Currently, considering that is the leading cause of death globally, breast cancer needs to be optimally investigated with a better data collection approaches, appropriate healthcare facilities, preventive strategies, and deep and thorough research on designing efficient theranostics.

The preliminary detection of breast cancer using modern methods includes self-diagnosis, medical check-ups, radiography, positron emission tomography (PET), mammography, and magnetic resonance imaging (MRI).^6^ The primary management of breast cancer historically relied on surgical intervention, including lumpectomy, partial mastectomy, and complete mastectomy, complemented by adjuvant radiation therapy to mitigate the risk of recurrence. These treatment modalities are selected based on the tumor characteristics, stage of the disease, and patient factors, aiming to optimize the therapeutic outcomes, while preserving the breast tissue to the greatest extent possible. Adjuvant therapeutic modalities, comprising endocrine therapy for hormone receptor-positive neoplasms, cytotoxic chemotherapy aimed at eradicating fast-proliferating malignant cells, and immunotherapy designed to leverage the capacity of the host immune system to combat oncogenic cells, are systematically integrated in treatment regimens to optimize the therapeutic efficacy and ameliorate prognostic indices in patients. These interventions are strategically deployed based on the oncogenic phenotype and individual patient profiles to mitigate the recurrence risk and augment survival probabilities by targeting specific molecular and cellular pathways implicated in tumor development and progression. However, despite these advancements made in interpreting the molecular functionality for diagnosing carcinogenesis and tumor development, and offering molecular targeted therapies, these theranostics approaches have some major limitations that need to be resolved, such as the destruction of normal cells, attaining insufficient tumor site drug dosage, and medicine instability. For instance, the universally used chemo-therapeutic drugs, 5-fluorouracil and doxorubicin, induce leukopenia, cardiotoxicity, myelotoxicity, and renal toxicity. Toxicity in the bladder, cutaneous, and pulmonary is also induced by the combinatorial utilization of cyclophosphamide and bleomycin.^7^ Thus, the development of effective therapies and rigorous diagnosis procedures remains a significant priority. Currently, nanotechnology has shown noteworthy prospects to improve cancer therapy with newer treatment and diagnosis approaches. The utility of nanotechnology is underscored by its diverse applications across multiple disciplines, including medicine, toxicology, biology, chemistry, pharmacology, engineering, mathematics, and materials science. It is projected to drive significant advancements, with its valuation often predicated based on its attributes such as a precise size range, targeted delivery capabilities, enhanced biocompatibility, and minimal toxicological impact. These characteristics make nanotechnology a pivotal element in the development of innovative solutions and methodologies in these fields, promising substantial progress and breakthroughs in the understanding and manipulation of various biological phenomena at the nanoscale. Nanotechnology is aimed at “the generation of vital materials, appliances, and mechanisms that can be utilized to transform matter in the range of 1–100 nm.^7^ The transportation of nanoparticles with a size of 1–100 nm within cells and tissues is very efficient. Furthermore, their surface modification with appropriate substrates allows their specific targeting to the cells of interest and controlled drug release. Biocompatible and non-toxic NP carriers harbor new potential in gene therapy of cancer.^8^ Recently, some anticancer drugs based on nanotechnology with approval from the FDA, USA include DaunoXome® (Gilead Sciences, Foster City, CA, USA), Myocet™, Abraxane®, and Doxil®. Herein, we provide an in-depth analysis of different nanotherapeutics that utilize particles with nanoscale dimensions and their strategic implementation in the management of breast cancer. Additionally, it traces the oncogenic progression specific to breast cancer and delineates the biochemical mechanisms by which metallic nanoparticles facilitate cytotoxic responses within the breast.

## 2. Pathophysiology of breast cancer

Cancer is initiated by the abnormal proliferation and division of cells, which is mainly attributed to the mutation of regulatory genes.^9^ The genes involved in cancer development and progression are comprised of three types, including oncogenes, proto-oncogenes, and tumour suppressor genes. Basically, proto-oncogenes are normal genes leading to no health-related abnormalities. However, when these proto-oncogenes mutate, they become oncogenes, which produces various onco-proteins, thereby causing abnormalities in the overall cell cycle and cancer. Oncoproteins disrupt cellular homeostasis, affecting cell cycle arrest, apoptosis, and genomic stability. By altering signalling pathways, they either promote unregulated cell growth or trigger programmed cell death. Moreover, their influence extends to compromising the mechanisms safeguarding genomic integrity, thereby accelerating mutagenesis and enhancing the propensity for cancerous transformation. The proteins coded by the tumour suppressor genes, also called molecular switches, are involved in DNA repair mechanisms and clearance of damaged cells. Their mutation also causes abnormalities in cell division and growth. Also, mutations in critical genes involved in cell cycle regulation pathways lead to the initiation of cancer development. The initiation process is accompanied by efficient DNA repair mechanisms, and thus the initiated cells do not die while progressing towards preneoplastic focal lesion development. The cells of the preneoplastic focal lesion show continuous proliferation due to the constant supply of factors promoting cancer development, ultimately leading to the development of metastasis.^9,10^ Presently, breast cancer accounts for the most prevalent malignancy worldwide. If it is identified in its early stage, it can be healed quite simply; however, when it becomes malignant, it drastically affects the survival rate of the patients. Cancer metastasis is divided into four distinct patterns including lung, liver, bone and brain. The benign to metastasis progression involves a cascade of events, namely, cell invasion from the basement membrane into the surrounding tissues, intravasation, extravasation, colonisation, and finally distant metastasis. Intravasation occurs *via* the disruption of junction proteins of endothelial cells aided by perivascular macrophages together with the tumour cells interacting with endothelial cells (ECs). Extravasation involves blood circulation among cell junctions of different endothelial cells with the aid of certain specific factors.^9^ The metastatic cells escaping dormancy in the blood vessels form micro-metastatic foci and start growing with the interaction of other cells. Breast cancer metastasis in the brain can be divided into three types depending on its anatomy, including leptomeningeal metastasis, parenchymal metastasis, and choroid plexus metastasis. The most common in the population is parenchymal metastasis, which is comprised of multiple metastases (78%) and solitary metastases (14%). Leptomeningeal metastasis is seen in approximately 8% of breast cancer patients, while choroid plexus metastasis is a rare event.^11^ The two main molecular targets of breast cancer are epidermal growth factor 2 (ERBB2) and estrogen receptor alpha (ERα). (ERα), involved in 70% of invasive breast cancer cases, is a transcription factor, and steroid receptor whose activation initiates the cascade of oncogenic pathways. Another closely related steroid receptor is the progesterone receptor (PR), which acts as biomarker for ER α-signalling. Its overexpression accounts for ∼20% breast cancers and shows poor prognosis.^12^

Breast cancer mortality predominantly results from metastasis rather than the primary tumor itself. CD44, a cell surface glycoprotein, is significantly expressed on cancer stem cells (CSCs), playing a pivotal role in breast cancer metastasis by facilitating invasion and adhesion processes. These CSCs, characterized by their high expression of aldehyde dehydrogenase (ALDH) 1, are capable of invading tissue and developing resistance to apoptosis, partly due to the role of ALDH1 in maintaining optimal levels of reactive radicals for these functions. Furthermore, elevated ALDH1 expression contributes to chemoresistance and metastatic progression in breast cancer through the activation of cell-signaling pathways, including Notch, Wnt/β-catenin, and the hypoxia-inducible factor (HIF)1α/vascular endothelial growth factor (VEGF) pathway, which promote the stem-like properties of these cells. HIF1α is instrumental in modulating the metastatic microenvironment of CSCs by diminishing mitochondrial oxidative stress and augmenting the synthesis of antioxidants, which are mechanisms that underlie the development of chemoresistance. In concern with related genes such as VEGF and TWIST (Twist related protein), HIF1α stimulates angiogenesis and activates matrix metalloproteinases (MMPs), as well as regulates the expression of E-cadherin and β-catenin, crucial factors in the metastatic cascade. Triple-negative breast cancer (TNBC), which accounts for approximately 20% of overall breast cancer cases, is characterized by its aggressive nature, high propensity for early recurrence, and metastasis. The absence of targetable hormone receptors in TNBC limits therapeutic options, rendering chemotherapy the primary modality of first-line treatment. However, the systemic administration of chemotherapy is associated with significant adverse effects and a heightened risk of mortality, underscoring the urgent need for alternative therapeutic strategies. In this context, the development of novel nanotherapeutic approaches, including photothermally targeted near-infrared (NIR)-responsive nanomedicine that induces immunogenic cell death, presents a promising avenue for effective TNBC treatment. These combinational therapies aim to minimize side effects and improve patient outcomes, highlighting the critical importance of continued research in this area to address the challenges posed by this formidable subtype of breast cancer.^13^

## 3. Conventional therapies for breast cancer

The primary goal of therapeutics for non-metastatic breast cancer is the removal of tumor from the breast and surrounding lymph nodes as well as the prevention of its metastatic recurrence. Nonmetastatic cancer can be treated locally. Breast cancer treatment includes surgical resection and axillary lymph node removal, combined with postoperative radiation treatment. Systemic therapy may be used prior to surgery (neoadjuvant), either postoperatively (adjuvantly) or both. The subtype of breast cancer regulates the overall treatment. Standard systemic therapy consists of endocrine therapy and is being used to treat all HR+ tumors with chemotherapy. Trastuzumab-based ERBB2 (epidermal growth factor receptor gene)-directed Ab-therapy combined with chemotherapy is also utilized for all ERBB2+ tumors. Currently, chemotherapy represents the only treatment possibility for triple-negative breast cancer. The therapeutic goals for metastatic breast cancer are to prolong life and counteraction of symptoms. Presently, metastatic breast cancer remains a terminal illness in almost all affected patients. Some common therapies for breast cancer modalities are discussed below.^12,14^

### 3.1. Hormonal therapy

Breast cell growth is regulated by estrogen receptors (ER) in response to estrogen. The human ER protein has 595 amino acids and a molecular weight of 66 kDa, forming six different functional domains, including estrogen and DNA binding domains. ER protein functions as a transcription factor belonging to the superfamily of nuclear hormone receptors, which is responsible for the initiation of the transcription estrogen response element, (ERE). Estrogen is produced by the ovary and acts as a ligand for ER by binding to it upon its diffusion through the endoplasmic reticulum. Upon the binding of estrogen, the estrogen receptor dimerizes and translocates into the nucleus for binding with the promoter region of ERE, causing downstream gene expression. Thus, premenopausal women show strong regression even in the cases of advanced breast tumors upon the removal of ovaries. Tamoxifen, raloxifene and arzoxifene are recognized as selective estrogen receptor modulators (SERMs), which through their AF2 domain, elicit the effects of ER activation.^15^ Around 75% of all breast cancers involve hormone receptors, and thus hormonal therapy has revealed a remarkable reduction in reoccurrence of breast cancer and increased the lifespan of patients by 10 years. Adjuvant tamoxifen treatment for five years reduced the annual breast cancer death rate by 31%.^15^

### 3.2. Immunotherapy

The tyrosine kinase receptor human epidermal growth factor receptor 2 (Her2) acts as a molecular switch for breast tumors. In 25% of breast tumors, it is upregulated due to aberrant gene amplification. Dimerization of Her2 is crucial for signaling cascade activation promoting the survival of the cells *via* the Ras–Raf–mitogen-activated protein kinase, ERK kinase (MEK)/ERK pathway. Consequently, this discovery aided in the discovery of trastuzumab, which is the first targeted anti-kinase therapy based on genomic research. The Food and Drug Administration (FDA) approved trastuzumab for invasive breast cancer therapy involving the overexpression of Her2.^16^

### 3.3. Chemotherapy

A “receptor-negative” or “triple negative” category refers to cancers that lack the expression of three receptor proteins, namely, PgR, ER, and ERBB2 receptors.^17^ This breast cancer subtype makes up about 10–15% of breast cancer cases and is regarded as one of the most extensively proliferative and aggressive types of cancer with poor prognosis.^18,19^ Currently, standard chemotherapy is the only management option in the case of triple negative cancer.^19^ Chemotherapy together with taxanes namely paclitaxel and docetaxel are very efficient cytotoxic agents. However, despite the use of these drugs and therapies, the average survival of metastatic breast cancer patients is still ∼18 months. The tumor microenvironment and breast cancer stem cells are prominently responsible for the failure of chemotherapy and the increase in disease resistance.^20^ The limited effectiveness of chemotherapy can be partly attributed to the lack of an optimal dosage of drugs in an attempt to limit chronic toxicities and side effects.^7^

## 4. Limitations of conventional therapies

Considering that breast cancer is currently treated upon employing a combination strategy comprised of chemotherapy and adjuvant therapy despite their established efficacy, the determination of the side effects and risks associated with these therapies is difficult. Consequently, these treatments can be lethal mostly owing to the killing of non-targeted cells. Given that cytotoxic agents are mostly non-selective in their activity, they damage healthy replicating cells even in the gastrointestinal epithelia, bone marrow, and hair follicles. Furthermore, although targeted therapies show a significant decisive effect as evident by multiple clinical studies, they also pose considerable side effects. Also, the diagnostic and prognostic statistics provided in different regions are specific to the populations of women of that nationality.^7^

For instance, methotrexate (MTX), a chemotherapeutic agent and folic acid analog, is utilized in the breast cancer treatment due to its capacity to inhibit cell proliferation. Its anticancer activity is mediated through the inhibition of dihydrofolate reductase (DHFR), an enzyme critical for the biosynthesis of DNA, RNA, thymidylate, and proteins within the cell. However, the Biopharmaceutical Classification System categorizes MTX as Class III, highlighting its high solubility but low permeability due to its suboptimal systemic absorption. Consequently, MTX exhibits a limited therapeutic index and is associated with adverse effects, including hepatic and gastrointestinal toxicities such as nausea, abdominal discomfort, and stomatitis. These limitations underscores the need for innovative delivery systems, such as a multifunctional nanomedicine platform that facilitates the co-delivery of methotrexate and mild hyperthermia, to enhance the efficacy and safety profile of breast cancer therapy.^21^ Another compound that has shown promising abilities to be employed as an anticancer agent is evodiamine (EVO). It is basically an indolequinone alkaloid extracted from *Evodia rutaecarpa*, a multi-purpose herb native to China.^22^ The mechanism of action of EVO includes the activation of caspases, which are critical enzymes responsible for executing apoptosis.^22^ However, despite its potent anticancer efficacy, the clinical application of EVO is hindered by its limited pharmacokinetics, including poor aqueous solubility, diminished gastrointestinal absorption, and reduced oral bioavailability.^22^ Additionally, EVO administration is associated with adverse effects ranging from gastrointestinal disturbances (diarrhoea, constipation, anorexia, and stomach discomfort) to severe toxicities (hepatotoxicity and cardiotoxicity) under high doses or prolonged usage.^22^ Thus, addressing these challenges necessitates the exploration of advanced drug delivery systems, such as nanotechnology-based carriers, which hold potential to enhance the bioavailability and therapeutic index of EVO, thereby mitigating its side effects and unlocking its full pharmacological potential. Doxorubicin, approved by the FDA in 1974, is another highly utilized anticancer agent, which is recognized for its advantageous pharmacokinetic attributes, including minimal plasma concentration and extensive tissue distribution/penetration post intravenous (IV) administration. These properties contribute to its broad-spectrum anticancer effectiveness. Nonetheless, a significant limitation of doxorubicin is its pronounced accumulation in cardiac tissue, resulting in a predisposition to cardiomyopathy, a severe adverse effect. Thus, to mitigate this cardiotoxicity, the development of ‘Doxil,’ a nanoformulated liposome-encapsulated version of doxorubicin, has been adopted to enhance its therapeutic efficiency.^23^ Doxil demonstrates a notable increase in tissue distribution, particularly in the epidermis and dermis, and exhibits enhanced uptake by spindle cells, a mechanism not solely reliant on the enhanced permeability and retention (EPR) effect of the tumor.^23^ Consequently, Doxil offers superior efficacy in treating specific cancers, such as ARKS4, compared to conventional doxorubicin.^23^ Although Doxil may increase the incidence of hand-foot syndrome, its reduced cardiac penetration significantly lowers the risk of cardiomyopathy, presenting a balanced profile of enhanced anticancer potency and diminished cardiotoxicity.^23^ Paclitaxel is a prominent anticancer agent, but is associated with significant formulation challenges due to its extremely low solubility in water (<0.01 mg mL^−1^) and most pharmaceutically acceptable solvents.^24^ The formulation known as Taxol, a 50 : 50 mixture of paclitaxel and Cremophor EL, utilizes Cremophor EL as a solubilizing agent.^24^ However, this combination has been linked to severe allergic reactions. Pharmacokinetic analysis of Taxol indicates a high plasma concentration but limited tissue distribution/penetration to the target organs, potentially diminishing its therapeutic effectiveness against certain solid tumors.^24^

## 5. Pristine characteristics of nanomaterials for breast cancer therapy

The implementation of non-invasive diagnostic screening techniques, such as mammography and echography, has significantly improved the five-year relative survival rate for women diagnosed with breast cancer (BC) from 75% in the 1970s to 91% in the mid-2010s.^25^ However, despite these advancements, the prognosis for patients identified with advanced-stage BC using these methodologies remains dismal, with a life expectancy as low as 26%.^25^ The primary contributors to mortality in these cases are metastases to critical sites such as lymph nodes, lungs, liver, bones, and brain.^25^ Consequently, there is an urgent requirement for the development of more effective breast cancer treatments that extend beyond the conventional modalities of chemotherapy, radiation, and surgical interventions to address the needs of patients in the current landscape. Nanomaterials and nanotechnology are not strangers to oncology. Liposomes are the earliest examples of nano-based cancer therapies. Conventional chemotherapy and immunotherapies utilize non-selective mechanisms that in addition to eradicating malignant cells, also compromise the viability of a multitude of normal cells. This collateral damage results in a spectrum of adverse effects, some of which can be severe and potentially fatal.^26^ Nanomedicines are emerging as a forefront therapy for cancer due to their several key advantages, as follows: (1) increased drug accumulation in tumors *via* the enhanced permeability and retention (EPR) effect, augmenting anticancer efficacy. (2) Prolonged systemic circulation and elevated plasma concentration of nanomedicines, reducing their clearance by the reticuloendothelial system (RES) and minimizing toxicity through decreased drug deposition in healthy organs. (3) The EPR effect coupled with extended systemic circulation allows for the development of a universal nanodelivery platform capable of administering various anticancer agents. The blood–brain barrier makes the systematic treatment of breast cancer less fruitful compared to peripheral metastasis. Thus, improving the penetration of existing medicines can serve as an alternative approach for efficient treatment. Nanoparticle-therapeutic drug conjugates delivered intravenously are highly efficient systems for targeting specific overexpressed antigens or receptors involved in cancer development.^27^ Nanocarriers are potentially highly efficient for drug delivery owing to their remarkable properties of large drug-loading capacity and protection of the loaded drugs from fast clearance. Nanocarriers build a gradient, allowing drugs to diffuse in a controlled manner through the tumor micro-vessels. This property of controlled and passive diffusion of drugs is called the EPR (enhanced permeability and retention) effect of nanoparticles. Nanocarriers also overcome the issue of transporter efflux.^28^

Moreover, the strategy of extending the systemic circulation time through nanoparticle surface modification (*e.g.*, PEGylation) has shown reduced nonspecific RES uptake and toxicity in mouse models. However, this approach may not uniformly benefit human cancer treatments, potentially disrupting the drug distribution across organs and affecting its safety profiles. Additionally, the concept of a universal nanodelivery platform faces limitations in clinical efficacy, as evidenced by drug-specific outcomes, for example, liposomal encapsulation reduces the cardiotoxicity of doxorubicin (Doxil) but compromises the effectiveness of paclitaxel. Conversely, albumin-bound paclitaxel nanoparticles (Abraxane) enhance its distribution and efficacy in treating lung, pancreatic, and breast cancers, whereas the same strategy may increase cardiotoxicity of doxorubicin.^29^ These observations underscore the necessity for developing drug-specific nanodelivery systems tailored according to the physicochemical, pharmacokinetic, and pharmacodynamic profiles of each anticancer agent to optimize their therapeutic efficacy and safety.^30^

Despite the challenges associated with the modest success rate of nanomedicines in human cancer treatments, particularly in clinical trials, there remains substantial evidence that nanoparticles and nanocarriers possess the potential to significantly advance cancer therapy, which is contingent on their application being grounded in comprehensive and meticulous research. Lipid-based nanoparticles, polymeric nanoparticles, and inorganic nanoparticles represent the forefront of desirable nanotechnological approaches due to their enhanced capability for the targeted delivery of therapeutic agents such as nucleic acids, chemotherapeutics, and immunotherapeutics directly to cancerous cells.^31^ This targeted delivery potential underscores the transformative promise of nanomedicine in oncology, provided that the strategies for their development and application are rigorously validated through extensive research. Carbon nanotubes are widely used in DNA mutation detection and as a biomarker for the detection of diseased proteins. Dendrimers are utilized for target sequestration, image contrast agent and controlled drug delivery.^31^ Nanocrystals help in improving drug formulations that are poorly soluble. Nanoparticles play a role in multifunctional therapies and as permeation enhancers, agents for apoptosis and angiogenesis and MRI contrast agents. Similarly, nano-shells are utilized for deep tissue tumor cell thermal ablation and tumor imaging.^32^ Before discussing the potential breakthroughs facilitated by nanotechnology, it is necessary to present an overview of current nanotechnology.^27,33^ Clinically, polymer nanoparticles are employed for the treatment of breast cancers. For instance, docetaxel is carried by polymer–lipid nanoparticles (polysorbate (PS)-80) to brain metastatic lesions, which were shown to be efficacious in mouse model therapy and enhanced their median survival.^34^ Mimicking low density lipoproteins (LDL), PS-80 coated nanoparticles are being explored for LDL-receptor mediated transcytosis, which is a newly developed approach for drug delivery utilizing the interaction between antibody-drug conjugates and their respective receptors.^34^ This leads to endogenous lipidation in association with apolipoprotein E, thereby, facilitating their passage across the BBB.^33^

In recent years, metallic NPs with anti-cancer properties have been developed *via* the green synthesis approach utilizing the phytochemicals present in plants (Table 1).

**Table 1:** List of green synthesized nanoparticles exhibiting anticancer activity^10^

Plant	Plant part	Nanoparticle shape and size	Anticancer activity
*Alternanthera sessilis*	Shoots/aerial parts	Silver NPs	3.04 μg mL^−1^
		Spherical/10–30	
*Andrographis echioides*	Leaf	Silver NPs	31.5 μg mL^−1^
		Pentagonal, cubic, hexagonal/68.06	
*Butea monosperma*	Leaf	Silver NPs	Dose dependent
		Spherical/20–80	
*Citrullus colocynthis*	Root	Silver NPs	2.4 μg mL^−1^
		Spherical/7.39	
*Citrullus colocynthis*	Fruit	Silver NPs	>30 μg mL^−1^
		Spherical/19.26	
*Citrullus colocynthis*	Leaf	Silver NPs	>30 μg mL^−1^
		Spherical/13.37	
*Citrullus colocynthis*	Seeds	Silver NPs	>30 μg mL^−1^
		Spherical/13.37	
*Olax scandens*	Leaf	Silver NPs	Dose dependent
		Spherical/30–60	
*Piper longum*	Fruit	Silver NPs	67 μg mL^−1^
		Spherical/46	
*Rheum emodi*	Root	Silver NPs	Dose dependent
		Spherical/27.5	
*Syzygium cumini*	Flower	Silver NPs	Dose dependent
		Spherical/40	
*Taxus baccata*	Needles	Silver NPs	37 μg mL^−1^
		Spherical/56	
*Ulva lactuca*	Whole plant	Silver NPs	37 μg mL^−1^
		Spherical/56	
*Sesbania grandiflora*	Leaf	Silver NPs	20 μg mL^−1^
		Spherical/22	
*Mimosa pudica*	Leaf	Gold NPs	6 μg mL^−1^
		Spherical/12	
*Musa paradisiaca* (banana)	Stem	Gold NPs	—
		Spherical/30	
*Antigonon leptopus*	Aereal parts	Gold NPs	257.8 μg mL^−1^
		Spherical, triangular/13–28	
*Corallina officinalis*	Aqueous extract	Gold NPs	—
		Spherical/14.6	
*Phoenix dactylifera*	Flowers	Gold NPs	4.76 μg mL^−1^
		Near spherical/95	
*Vitis vinifera*	Aqueous extract	Gold NPs	—
		Spherical/20–45	
*Acalypha indica*	Leaf	Gold NPs	—
		Spherical/20–30	
*Tabernaemontana divaricata*	Leaf	Zinc NPs	30.6 μg mL^−1^
		Spherical/36 ± 5	
*Tabernaemontana divaricata*	Leaf	Zinc NPs	30.6 μg mL^−1^
		Spherical/36 ± 5	
*Tabernaemontana*	Leaf	Zinc NPs	30 μg mL^−1^
		Spherical/36 ± 5	
*Borassus flabellifer*	Leaf	Zinc NPs	0.125 μg mL^−1^
		Spherical/55	
*Embelia ribes*	Root	Zinc NPs	9.62 ± 1.9 μg mL^−1^
		Spherical/130–150	
*Saccharum officinarum*	Juice	Zinc NPs	16.7 ± 0.5 μg mL^−1^
		Spherical/19 ± 2.3	
*Anabaena variabilis*	Pigment	Zinc NPs	16.5 1.6 μg mL^−1^
		Spherical/42 ± 3	
*Atropa belladonna*	Leaf	Zinc NPs	12 ± 0.9 μg mL^−1^
		Hexagonal/34 ± 3.2	
*Azadirachta indica*	Leaf	Copper NPs	27.4, 45.3
		Spherical/12	37 μg mL^−1^
*Olea europaea*	Leaf	Copper NPs	1.47 μg mL^−1^
		Spherical/20–50	
*Acalypha indica*	Leaf	Copper NPs	56.16 μg mL^−1^
		Spherical/26–30	

The green synthesis of metal and metal–oxide NPs (silver nanoparticles (AgNP), gold nanoparticles (AuNP), copper oxide (CuONP), zinc oxide nanoparticles (ZnONP), *etc.*) has opened the door for highly efficient treatment and medication of cancer.

For precision in the field of therapeutics and diagnostics, nanomaterials are becoming important medical tools owing to their functionality.^35–40^ Scientists have developed barcoded nanoparticles, such as barcoded liposomes, carrying a diagnostic agent (indocyanine green, (ICG)), which can be injected intravenously for real-time imaging. Barcoding of nanoparticles facilitates real-time imaging and tracking of their activity and biodistribution. Three barcoded nanoparticles containing chemotherapeutics such as cisplatin, doxorubicin, and gemcitabine have been clinically approved, which can be utilized to predict the therapeutic potential of drugs and charter greater gains in the field of personalized medicine.^41,42^

The new nanodrug delivery system (NDDS) discovered (*in vitro*) by scientists combines an anti-metastatic drug (silibinin) and photothermal agent ICG (indocyanine green), which has been shown to prevent metastasis and tumor growth simultaneously. In short, ICG and silibinin are self-assembled into polycaprolactone (PCL) lipid nanoparticles, Pluronic copolymer F68, and soybean phosphatidylcholine (SPC). SIPN is effective in inducing a combinational therapeutic effect of anti-metastatic drugs and photothermic agents upon NIR laser irradiation to inhibit the growth of cancer cells, as confirmed from the *in vitro* assays.^5^ This novel drug delivery system was designed to overcome the poor water solubility of silibinin. It also aimed to achieve a more rapid photothermal-induced release of the drugs in tumor cells cooperatively to inhibit the growth and metastasis of cancerous cells *in vitro*.

**Fig. 2:**
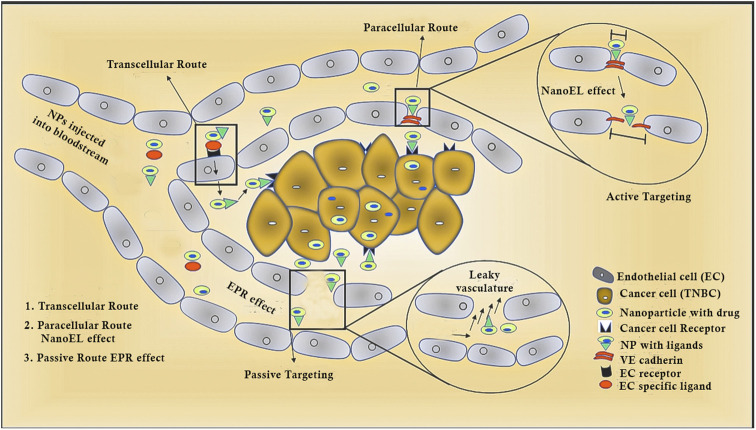
Diagrammatic representation of different routes followed by nanoparticles.^45^

Protein-based nanosystems, specifically albumin nanoparticles, have attracted significant attention due their inherent advantages, including their non-immunogenic nature, bio-stability, biodegradability, high drug-binding capacity, biocompatibility, low toxicity, and extended circulation times.^43^ Among them, bovine serum albumin (BSA) stands out as a nano-carrier for the delivery of various bioactive molecules and pharmaceuticals, which is attributed to its widespread availability, cost-effectiveness, and straightforward purification process.^43^ Evodiamine (EVO)-loaded BSA nanoparticles (ENPs) were synthesized *via* the desolvation technique, demonstrating enhanced anticancer activity by specifically targeting apoptotic pathways. Additionally, a notable increase in p53 and Bax levels, together with a decrease in Bcl-2 expression was observed through mRNA expression analyses and western blotting. Fluorescence microscopy revealed the improved cellular uptake of ENPs over free EVO, with ENPs significantly inhibiting colony formation and inducing apoptosis more effectively.^44^ Enhanced cytotoxicity and apoptosis induction, with cells arrested at the G2/M phase, were reported in MDA-MB-231 and MCF-7 breast cancer cells, indicating the potential of ENPs as a strategic approach for breast cancer treatment. In another study, hyaluronan (HA)-polyaniline (PANi)-imiquimod (R837) nanoparticles (HA-PANi/R837 NPs) were developed, exhibiting a high extinction coefficient and efficient photothermal conversion, making them suitable photothermal agents (PTAs) for targeted CD44-mediated photothermal ablation of triple-negative breast cancer (TNBC) tumors. R837, acting as a toll-like receptor 7 agonist, enhances the immune response against tumors.^44^ These nanoparticles facilitate tumor destruction *via* NIR-triggered photothermal ablation, promoting the release of tumor-associated antigens and synergizing with anti-CTLA-4 to activate immunogenic cell death (ICD), effectively eliminating residual tumor cells in mice and fostering an active immune memory to prevent relapse and metastasis.^44^ HA-PANi/R837 NPs, characterized by their non-toxicity, non-immunogenicity, biocompatibility, biodegradability, and high biosafety, leverage HA for targeted drug delivery, improved stability, prolonged circulation, and enhanced CD44-mediated intracellular uptake, culminating in efficient therapeutic outcomes (Fig. 2).^44^

### 5.1. Nanomaterial design criteria

Despite these promising preclinical outcomes in xenograft cancer models, the clinical translation of many anticancer nanomedicines has not met expectations. The discrepancy partly arises from the variability of the EPR effect between mouse xenograft models and human cancers, and the assumption that the EPR effect is directly correlated with improved drug accumulation. Clinical data revealed that nanomedicines do not consistently achieve superior drug accumulation in human tumors compared to free drugs, which is attributed to the heterogeneity of EPR across different tumors, patients, and organs within the same individual. This heterogeneity challenges the efficacy translation from preclinical models to patients. In preclinical testing, nanomedicines have demonstrated superior capacity to impede tumor proliferation and extend the survival of treated cells compared to conventional free drugs. However, in clinical trials, the predominant benefit of nanomedicines for patients is frequently the mitigation or modification of adverse effects rather than enhanced efficacy. A critical factor in the development of effective nanomedicines involves the meticulous crafting of probes and protocols for stratifying patients for inclusion in clinical studies. The clinical and commercial viability of nanomedicines is significantly dependent on the application of rational design principles, underscoring the importance of strategic formulation and evaluation in advancing these therapeutic agents.^55^ Combinatorial therapy presents a promising approach for enhancing therapeutic outcomes by addressing the limitations inherent to monotherapies, thereby facilitating increased apoptosis in cancer cells. This strategy employs a unified nanosystem that leverages diverse physicochemical mechanisms to achieve a synergistic anticancer effect (Table 2).^56^

**Table 2:** Different types of nanoparticles with their targeting site and origin

S. No.	Nanoparticle formulation	Targeting site	Origin country/company	References
1	Abraxane (albumin-bound paclitaxel nanoparticles)	General tumor site	Celgene Corporation (United States)	46
2	Doxil (liposomal doxorubicin)	General tumor site	Johnson & Johnson (United States)	47
3	Onivyde (irinotecan liposome injection)	General tumor site	Merrimack Pharmaceuticals (United States)	48
4	MM-302 (liposomal doxorubicin with HER2-targeting antibody)	HER2-overexpressing breast cancer cells	Merrimack Pharmaceuticals (United States)	49
5	CALAA-01 (siRNA-containing cyclodextrin nanoparticle)	Specific gene targets in breast cancer cells	Calando Pharmaceuticals (United States)	50
6	BIND-014 (docetaxel-loaded polymeric nanoparticle)	General tumor site	BIND Therapeutics (United States)	51
7	Genexol-PM (polymeric micellar paclitaxel)	General tumor site	Samyang Corporation (South Korea)	52
8	Lipid-coated calcium phosphate nanoparticles	Breast tumor cells and metastatic sites	HiberCell (United States)	53
9	CT-2106 (polymeric nanoparticle paclitaxel)	General tumor site	CritiTech (United States)	54

In the context of targeted cancer therapy, LAPA (Lapatinib) exhibits suboptimal clinical efficacy in HER2-negative patient populations, which is primarily due to its limited oral bioavailability. However, it is beneficial for the precision targeting of the epidermal growth factor receptor (EGFR). Conversely, doxorubicin (DOX), despite its transformative impact on oncology, is marred by nonspecific systemic toxicity. Thus, addressing these pharmacological challenges, a novel nanomedicine has been engineered, encapsulating both LAPA and DOX within a glycol chitosan-stabilized nano-formulation. This nanoconjugate demonstrates a synergistic effect on triple-negative breast cancer (TNBC) cells, surpassing the efficacy achievable through the mere physical combination of the free drugs. The nanomedicine facilitates the sustained release of the therapeutic agents, inducing apoptosis and achieving approximately 80% cancer cell mortality, while exhibiting an enhanced safety profile in Balb/c mice, markedly reducing the cardiotoxicity typically associated with DOX. The combined nanotherapeutic approach not only suppresses the growth of primary 4T1 breast tumors but also significantly curtails metastasis to vital organs such as the lungs, liver, heart, and kidneys, outperforming the monotherapies. These initial findings underscore the potential of this combinational nanomedicine as a formidable strategy for the treatment of metastatic breast cancer.^56^ A multifunctional core–shell nanoparticle system, integrating a molybdenum disulfide core with a barium titanate shell (MoS_2_@BT), was engineered to deliver dual therapeutic modalities, photothermal therapy and chemotherapy, specifically targeting the folate receptor to enhance treatment effectiveness against triple-negative breast cancer (TNBC) MDA-MB-231 cells.^57^ This nanosystem was further functionalized with polydopamine (PDA), and subsequently modified with folic acid (FA) for improved stability and targeted tumor cell engagement, resulting in the formulation designated as MoS_2_@BT-PDA-FA (MBPF).^57^ The encapsulation and controlled release rates of gemcitabine (Gem) by MBPF were determined to be 17.5 wt% and 64.5.%, respectively. MBPF demonstrated a notable photothermal conversion efficiency (PCE) of 35.3%, together with superior biocompatibility, as confirmed by MTT assays. Upon near-infrared (NIR) laser exposure, MBPF efficiently raised the local temperature to 56.0 °C, facilitating targeted Gem release within TNBC cells. Leveraging its dual-action therapeutic strategy, MBPF significantly reduced TNBC cell viability to 81.3%, showcasing its potent synergistic effects and marking an innovative advance in cancer treatment paradigms.^57^ An innovative strategy in nanomedicine design for cancer treatment involves the use of prodrug-based nanomedicines, which require activation by specific stimuli to exert antitumor effects. These nanomedicines are categorized into prodrug-encapsulated nanomedicines, polymer-conjugated prodrug nanomedicines, and self-assembled small molecule prodrug nanomedicines.^57^ In polymer-conjugated prodrug nanomedicines, drugs are covalently linked to polymers, forming prodrug-based nanoparticles. The copolymers used in these conjugates offer numerous functional sites for drug attachment, enhancing the drug loading efficiency through the cross-linking of anticancer molecules. Polymers such as polypeptides, poly(ethylene glycol) (PEG) block copolymers, polysaccharides, and poly amino acids provide active functional groups (hydroxyl, carboxyl, amino) suitable for constructing polymer-drug conjugate nanomedicines. Self-assembled small molecule prodrug nanomedicines, characterized by low molecular weight and nanostructure size, are formed through self-assembly and cross-linking processes. These nanomedicines, including pillararene-drug conjugates, peptide prodrugs, and lipid-drug conjugates (*e.g.*, squalenoylations, cholesteryl, and cyclodextrin-drug conjugates), offer a high drug loading efficiency. However, despite their advantages, they have certain limitations such as short circulatory half-life and structural instability. Thus, to address these issues, long amphiphilic chains such as DSPE-PEG are utilized to modify small molecule prodrug conjugates, enhancing their stability and circulation time.

Prodrug-encapsulated nanomedicines leverage nanocarriers (*e.g.*, inorganic nanoparticles, liposomes, micelles, and nanogels) to encapsulate anticancer prodrugs *via* noncovalent interactions. This approach provides improved targeting efficiency, drug utilization, and reduced side effects, although the drug loading efficiency remains a challenge, limiting clinical advancement. Despite being less efficient compared to other prodrug strategies, the flexibility of carrier choice has attracted significant interest in prodrug-encapsulated nanomedicines, highlighting their potential in cancer therapy innovation.^58^ Abraxane, also known as nanoparticle albumin-bound paclitaxel (nab-paclitaxel), represents a significant advancement in nanomedicine, demonstrating promising clinical efficacy. This formulation is created through the noncovalent association of paclitaxel with processed human serum albumin nanoparticles. Abraxane achieves notably superior tissue penetration of paclitaxel, up to nine times greater than that observed with conventional solvent-based formulations. Additionally, it exhibits a 33% increase in intra-tumoral drug concentration, a tenfold enhancement in the peak concentration of free paclitaxel, and a fourfold reduction in the rate of drug elimination. The clinical effectiveness of Abraxane has been validated through rigorous trials, including the GeparSepto study, which enrolled 1229 women with previously untreated primary invasive breast cancer, either unilateral or bilateral. This trial demonstrated that substituting solvent-based paclitaxel with nab-paclitaxel led to a significantly improved pathologic complete response rate following anthracycline-based chemotherapy. These findings strongly support the preferential use of Abraxane over traditional solvent-based paclitaxel formulations, underscoring its enhanced therapeutic profile and potential to improve treatment outcomes in breast cancer.^59^ In the domain of breast cancer (BC) nanotherapeutics, protein, liposomal, and polymeric nano-formulations prevail as approved interventions. However, their clinical efficacy frequently exhibits a limited reduction in toxicity without substantial enhancements in efficacy compared to conventional free drug formulations. Notably, Doxil, the first FDA-approved nanodrug (1995), represents a PEGylated liposomal formulation of doxorubicin, boasting a diameter of approximately 85 nm. Nonetheless, its utilization is tarnished by the notable risk of inducing congestive heart failure.^60^

Metallic NPs elicit comparatively more toxicity to cancer cells than normal cells. The cytotoxicity of metallic NPs to cancer cells is attributed to their ability to generate reactive oxygen species (ROS), caspase-3 activation, permeabilization of the outer membrane of mitochondria, and cleavage of DNA. These activities lead to cancer cell apoptosis, autophagy, and necrosis. The route of entry and method of uptake of these NPs by cells are highly dependent on the size of the NPs.^61^ Smaller NPs follow receptor-mediated uptake upon interaction with the caveolin receptor on the cell membrane. In the cell, NPs take different routes to perform specific functions. Their release profile in the cytosol can be either direct interaction with proteins or *via* surface modification of lysosome–endosome complex prior to their release in the cytosol. Once inside the cell, NPs start generating ROS and metal ions, which bind to the SH group causing S–S bond breakage. This results in malfunctioning of cell physiology, damaging the signalling pathways, ultimately causing apoptosis. Autophagy is also induced by NPs by causing disrupted protein aggregation, resulting in oxidative stress, organelle stress, and alteration in genes. Studies have shown that metal NPs also lead to the expression of elevated levels of autophagic vacuoles *in vitro* in animal and human cells.^10^ The therapeutic role of some metal nanoparticles is discussed below.

## 6. Metallic nanoparticles for breast cancer

### 6.1. Silver nanoparticles

The characteristic surface chemistry of silver nanoparticles (AgNPs) and their wide biological roles, such as anti-parasitic, antimicrobial, and anti-cancerous, imparted by their synthesis *via* green synthesis routes have prompted scientists to focus on green-synthesized AgNPs for breast cancer treatment.^62^ It was reported by Sanpui *et al.* that AgNPs disrupt the membrane integrity and other cellular mechanisms, hence causing apoptosis in cancerous cells. The green synthesis of AgNPs involves the utilization of a wide array of plant parts with brilliant therapeutic properties. Sugumari Vallinayagam *et al.* showed in their study that the 20–40 nm-sized AgNPs fabricated with *Naringi crenulata* leaf extract (NC-AgNP) affected the cellular function of cancer cells by inhibiting cell proliferation and deregulation of cell-cycle progression. The reduction of silver was achieved by the alkaloids, phenols, saponins, and flavonoids present in *Naringi crenulata*. NC-AgNPs when employed to treat HER2 positive breast cancer (SKBR-3) cell lines were shown to inhibit cell proliferation, deregulate the cell cycle progression, and hinder cell invasion. These observations indicated that NC-AGNPs treated breast cancer (HER2 positive) *via* HER2 inactivation, thereby inducing apoptosis and could also inhibit angiogenesis and metastasis simultaneously.^63,64^ The green synthesis of AgNPs with *Fagonia indica* (aqueous extract) was reported by Ikram Ullah *et al.* to induce anticancer properties. Recently, it was found that *Fagonia indica* exhibited anticancer properties in MCF7 cells by inducing their apoptosis. Basically, the as-synthesized AgNPs activate caspase 3, caspase 9 and ROS, thereby combinatorically *via* the cascade of cell signaling mechanism leading to endoplasmic reticulum stress, DNA damage, apoptosis, and protein misfolding. In the initial stage, DNA gets fragmented due to endonuclease activity, and this marks the prominent event in apoptosis. The phenolic and flavonoid components of *Fagonia indica* chelate and stabilize the AgNPs during their biosynthesis. It is evident in studies that *Fagonia indica* has significant anti-cancerous properties in MCF-7 cells. Apoptosis involves the expression of initiator caspases, such as caspase 9, and executioner caspases, such as caspase 3, which function as inactive zymogen in the cytoplasm and help in programmed cell death. AgNPs play a crucial role in activating caspases 3 and 9, simultaneously also generate ROS. These combined effects lead to damage of the endoplasmic reticulum and DNA, misfolding of proteins, and eventually apoptosis. Reports revealed that caspase 3 activation cleaves caspase-activated DNAse (CAD), causing DNA fragmentation, which causes apoptosis in the early cancer stages. The phenolic and flavonoid components of plants endow them with anticancer properties. These phytochemicals chelate and stabilize the synthesized nanoparticles, and simultaneously they also decrease the tumor volume. Furthermore, the biochemical pathways induced by silver nanoparticles that are responsible for the enhanced anticancer activities in MCF-7 cells include *via* mitochondrial dysfunction, autophagy, cell cycle arrest, and lipid peroxidation.^65,66^

### 6.2. Gold nanoparticles

Wide varieties of plants have been employed for the biological synthesis of gold nanoparticles (AuNPs). Fatemeh Yousefmehr *et al.* reported the synthesis of gold and reduced graphene oxide nanoconjugate (Au/rGO NC) utilizing the stem extract of *B. oleracea* plant. It exhibited photothermal therapy on MCF7 breast cancer cells.^66^ The MTT assay and DAPI staining confirmed the photothermal-based treatment of MCF7 breast cancer cells. The ROS generation activity by the Au/rGO NC was shown *via* the DPPH assay. The ability of Au/rGO NC to undergo light-to-heat conversion in the NIR light region of the spectrum is the prime reason for its therapeutic activities even at a very low concentration. The combination of Au/rGO NC and laser irradiation was reported to show maximum cell death (40.12%). The important properties of that make the Au/rGO synthesized from the stem extract of *B. oleracea* invaluable for breast cancer treatment (MCF7 breast cancer cells) are their non-toxic nature, amazing reducing and stabilizing ability, and excellent biocompatibility. Mohamed Hosny *et al.* reported the single-step efficient synthesis of AuNPs using halophytic plants, namely *Atriplex halimus* and *Chenopodium ambrosioides*. The spherical-shaped AuNPs with a size of 2–10 nm from *A. halimus*, and 40 nm from *C. ambrosioides* inhibited the proliferation of MCF 7 breast cancer cells. The MTT assay showed the relatively higher cytotoxicity of *A. halimus*-based AuNPs than the *C. ambrosioides*-based AuNPs. It was suggested that luteolin, a flavonoid present in both plants, serves as both reducing and capping agents during the synthesis of AuNPs. The AuNPs synthesized utilizing these two plants were shown to interact with MCF7 breast cancer cells by disrupting the metabolic, and physiological processes, cell membrane integrity, hampering ATP synthesis, causing oxidative stress, obstructing electron transfer, and causing the cells to shrink and undergo apoptosis.^67^ Nihal Saad Elbialy *et al.* showed that curcumin when conjugated with noble metal nanoparticles such as gold exhibit enhanced bioavailability and stability. The curcumin-based AuNPs remained stable for a period as long as six months. Nihal Saad Elbialy *et al.*, showed in this study that 0.72 μg mL^−1^ of curcumin-based AuNPs inhibited cell proliferation and induced apoptosis in MCF7 breast cancer cells. Curcumin acts as both a stabilizing and anticancer agent, and both these qualities make the curcumin-based AuNPs a highly qualified candidate for breast cancer therapy.^68^ AuNPs bonding with polymers is facilitated by the presence of ester carbonyl group in the polymers, and hence polymer-decorated AuNP nanoconjugates facilitate controlled drug release and efficient anticancer activity, and also inhibit MCF-7 proliferation. The mechanisms for this inhibition can be attributed to deoxythymidine monophosphate synthesis and misincorporation of 5-fluorodeoxyuridine monophosphate in the DNA chain or incorporation of 5-fluorouridine monophosphate in the Pluronic copolymer F68 RNA chain.^69^ In the study by Balakrishnan *et al.*, they utilized AuNPs conjugated with quercetin, which is a flavonoid exhibiting anticarcinogenic properties. The AuNP-quercetin nanoparticles exhibited a considerable increase in *in vitro* apoptotic population with increased nuclear condensation in MCF-7 and MDA-MB-231 breast cancer cell lines.^70,71^

### 6.3. Copper nanoparticles

The clinical application of copper oxide nanoparticles (CuONPs) has been studied owing to their antioxidant, anticancer, antimicrobial, and drug carrying capacity.^72,73^ The anti-cancerous activity of CuONP is mostly attributed to the release of the Cu^2+^ ion from CuONP, which eventually binds to DNA, causing DNA damage, and hence cell death. It is based on the ROS generation mechanism, causing apoptosis. Neran Ali Thamer *et al.* reported the green synthesis of CuONP utilizing the leaf extract of *Cordia myxa* L., which possessed a toxic effect on MCF7 breast cancer cells. A concentration of 100 μg mL^−1^ CuONP gave the highest inhibition of MCF7 breast cancer cells (71.1%) when treated for 24 h. With the incubation time of 48 and 72 h, inhibition rate increased to 80% and 85.2%, respectively.^72^ In another study, CuONP was synthesized with the help of *Helianthus tuberosus* (Ht) extract. It was further enclosed within starch (ST), and conjugated with folic acid (FA), thereby, leading to the synthesis of FA-ST-HtCuONPs. FA-ST-HtCoONPs exhibited remarkable cytotoxicity in human breast cancer (MDA-MB-231) cells due to their ROS generation ability, leading to nuclear damage, lowering of the mitochondrial membrane potential, and stimulating apoptotic-related protein expression. In this case, the folate receptor-based endocytosis enhanced human breast cancer therapy.^68^

A nanoformulation of (diethyldithiocarbamate) DE was achieved *via* green chemically synthesized Cu_4_O_3_ NPs or zinc oxide NPs for enhanced treatment of metastatic breast cancer cells. The apoptotic activity of the unique nano complexes of DE was investigated against highly metastatic breast cancer cells (MDA-MB 231). They were also evaluated for their pro-oxidant-mediated apoptotic activity and inhibitory efficacy in an orthotopic metastatic breast tumor animal model. CD (DE modified with copper oxide) and ZD (DE modified with zinc oxide) nano complexes were synthesized with sizes of 156.5 nm and 206.5 nm, respectively, negative zeta potential, and morphology similar to their corresponding metal oxide NPs (semi-sphere and rods, respectively). CD NPs were reported to possess the highest antitumor effect in reducing mammary tumor volume and weight, and they also served as efficient apoptotic markers in mammary tumor and liver tissues. *In vivo* studies of CD NPs were mostly linked to their pro-oxidant activity, as evidenced by the strongest ALDH1A inhibition in MDA-MB 231 cells. The pro-oxidant activity imparting the anti-cancerous effect of DE was enhanced by metal oxide NPs and *vice versa*, as evidenced by the lower IC_50_ values of both nanocomplexes compared to DE or metal oxide NPs.

### 6.4. Iron nanoparticles

Magnetic iron oxide nanoparticles (MNPs) are extensively employed in clinical research for development in the healthcare sector. Their important features that make them special for the diagnosis and therapy of a wide array of diseases are that they can be efficiently controlled in the presence of an external magnetic field and their magnetic drug and gene delivery capability, high resolution imaging potential, hyperthermia property, and high cellular uptake. Ghassan M. Sulaiman *et al.* reported a green synthesis route for MNPs with the leaf extract of *Albizia adianthifolia*, which acted both as a reducing agent and protecting agent owing to the presence of phenolic and flavonoid compounds, also acting as stabilizing agents. They played cytotoxic roles in human breast (AMJ-13) and (MCF-7) cancer cell lines, which was confirmed by the comet assay. The DNA fragmentation induced by MNPs suggested the genotoxicity and mutagenicity in DNA of cancer cells, which when combined with ROS production caused oxidative stress in HL-7702 cells, leading to nuclear condensation, chromosomal DNA fragmentation, and eventually apoptosis of breast cancer cells. Multifunctional iron oxide NPs with linker molecules have been constructed to be utilized in high energy radiotherapy, chemotherapeutics and photodynamic therapy.^72^

Under the influence of an external magnetic field, SPIONs could exhibit targeted delivery and increase the accumulation of nanoparticles in the diseased tissue. They work by raising the temperature of the tumor to around 40–45 °C through the hyperthermia effect, which causes irreversible injuries such as protein denaturation, plasma membrane perturbations, and organelle swelling, eventually leading to apoptosis. Earlier studies reported the incorporation of MTX directly on chitosan-coated SPIONs for the purpose of active targeting and hyperthermic effect. However, this combination reduced the superparamagnetic efficiency of SPIONs. Therefore, the co-loading of the drug (MTX) and SPIONs in NLC was performed, which exhibited a particle size of 214 ± 3 nm, with no change in zeta potential (−12.6 ± 1.6 mV) compared to SPIONnm-NCL (−11.8 ± 0.4 mV). The obtained PDI values for all the NLC formulations were less than 0.1, indicating the homogeneity, narrow size distribution, and low tendency to aggregate. The results showed a remarkable enhancement in the encapsulation efficiency of MTX in MTX-SPIONs co-loaded in NLC, which was 75%, and drug loading (3.3%). The haemolysis rate determined by the hemolysis assay for this system was 1.6% ± 0.3%, which is significantly lower than 5% (the standard reference level for clinical trials).

### 6.5. Selenium nanoparticles

*Acinetobacter* sp. SW30 cell suspension-based synthesized selenium nanoparticles show anti-cancerous activity. However, although chemically synthesized selenium nanoparticles show significantly higher anticancer activity, because of their toxicity towards non-cancerous cells, the biological synthesis protocol for selenium nanoparticle-based anticancer agents is gaining more significance. The proteins present in bacteria are found to be an essential component for the reduction of selenium nanoparticles. Vetrivel Cittrarasu *et al.* reported the green synthesis of selenium nanoparticles (SeNPs) utilizing *Ceropegia bulbosa* tuber aqueous extracts, whose *in silico* studies revealed their enhanced stability towards breast cancer gene 2 (BRCA2). The Glide docking protocol was utilized for binding energy calculation between the SeNP-BRCA2 complexes, thereby indicating the presence of highly electronegative amino acids on the surface of the BRCA2 protein. They were shown to exhibit cytotoxic effects in MDA-MB-231 breast cancer cells and HBL-100 normal breast cells.^74^

### 6.6. Palladium nanoparticles

Pd-NPs exhibit efficient photocatalytic activity, high chemical and thermal stability, and remarkable optical and electronic properties, which allow them to undergo different reactions, such as C–C bond formation and oxidation. They are reported to inhibit the growth of cancer cells and bacterial cells. However, a major hurdle in the utilization of Pd-NPs in clinical settings is their thermodynamic instability, which results in the formation of aggregates. Thus, to overcome this challenge, green synthesis methods have been explored, which show a remarkable improvement, as reported in some studies. For instance, Hana Sonbol *et al.* utilized the extract of brown algae, *Padina boryana*, for the synthesis of Pd-NPs, which were reported to exhibit therapy for breast cancer. The compounds present in *Padina boryana* responsible for the reduction of Pd include flavonoids, fatty acids, polysaccharides, phenolics. The cell viability assay confirmed the inhibition of the proliferation of breast cancer MCF-7 cells. The miRNA of apoptotic genes was also reported to be highly expressed when treated with Pd-NPs, suggesting their potential utilization in clinical sector for therapy of breast cancer.^75^

### 6.7. Carbon quantum dots

**Fig. 3:**
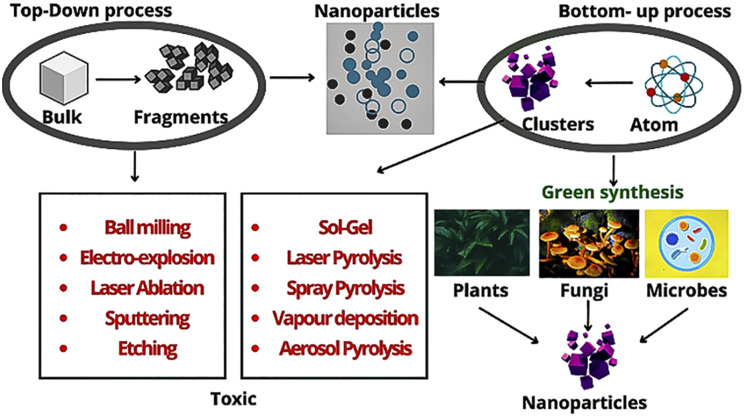
Different routes for the synthesis of nanoparticles.

*Aloe barbadensis* Miller (Aloe vera) extract was employed by Jalaja Prasad Malavika *et al.* for the green synthesis of carbon quantum dots (CQDs). The technique employed for this purpose was microwave-assisted reflux synthesis. The CQDs synthesized this way were reported to be internalized in cancer cells, showing blue emission in fluorescence microscopy. The CQDs in the case of human breast cancer cells showed anti-proliferative effects and exhibited excellent optical and fluorescence properties with brilliant water solubility and high quantum yield. The interaction of CQDs, synthesized especially through the green route of biomasses, with cancer cells resulted in ROS production, which destroyed MCF7 cancer cells. Also, they also simultaneously facilitated the bioimaging of these cells. The gel extract from the leaves of *Aloe vera* contains 75 pharmacologically active components, among which, aloe emodin inhibits the proliferation of human breast and cervical cancer cell lines and hepatocellular cancer cell lines.^76^ Also, walnut oil was shown to produce CQDs, exhibiting anticancer properties against MCF7 breast cancer cell lines (Fig. 3).^77^

## 7. Pre-clinical advancements of nanotechnology for breast cancer

Nanoparticle design, targeting strategies, efficacy, safety study and imaging are some key aspects in the preclinical development of nanomedicine for breast cancer. However, the literature suggests that in the eight phase III clinical trials comparing nanomedicines with free drugs (doxorubicin or paclitaxel), only two nanomedicines (in three phase III trials) have shown superior efficacy over free drugs (Doxil is superior to doxorubicin in AIDS-related Kaposi sarcoma and Abraxane is superior to Taxol in metastatic breast cancer). The other five nanomedicines in phase III studies did not reflect any significant differences in their efficacy compared to the free drugs, including Myocet *vs.* doxorubicin in metastatic breast cancer, Doxil *vs.* doxorubicin in metastatic breast cancer, Abraxane *versus* Taxol in gastric adenocarcinoma and Paclical in ovarian cancer. The latest study on the nanomedicines of paclitaxel (BIND-014,66 NK-10544) in phase III clinical studies (2016–2018) their elucidated poor clinical efficacies, questioning the future of nanomedicine research. The pre-clinicals trial of nanoparticle-mediated drugs are mentioned in Table 3.

**Table 3:** List of pre-clinical trial phases of nanoparticle-mediated drugs

Product name	Drugs	Trail phase
EndoTAG-1	Paclitaxel	Phase III
LEP-ETU (Liposomal Entrapped Paclitaxel-Easy To Use)	Paclitaxel	Phase II
LIPUSU®	Paclitaxel	Phase IV
Nal-IRI	Irinotecan	Phase I
Doxil® (US) Caelyx® (Europe)	Doxorubicin hydrochloride	Approved
Myocet	Doxorubicin citrate	Approved in Europe and Canada/phase III US
MM-302	Doxorubicin	Phase III
2B3-101	Doxorubicin	Phase II
Lipolatin (Regulon Inc.)	Cisplatin	Phase III
Mitoxantrone HCL liposome	Mitoxantrone	Phase II
ThermoDox®	Doxorubicin	Phase I/II

## 8. Limitations of nanomaterial-based cancer therapeutics

The pathway of therapeutic agents or nano-vectors and conventional formulations from the point of administration to the target region is thoroughly challenging. The extravasations of vascularly injected agents are hindered by various biological barriers such as tight junctions of epithelial cells and the BBB, often acting as a trigger for sensitization reactions. Early-generation dendrimers induced a weak antibody response, whereas a protein dendrimer conjugate was highly immunogenic. Similarly, fullerene antibodies recognized carbon nanotubes.^33^ Thus, indicating countermeasures for the prevention of sensitization to nanoparticle-enhanced therapy must be given a great deal of attention. However, the relevant issues stretch beyond considerations of biocompatibility, biodistribution and their production protocols. The key difficulty emerges because these nano-based theranostic approaches must pass three Food and Drug Administration (FDA) regulatory agencies: pharmaceuticals, medical devices, and biological agents. Consequently, they must be assessed from all three perspectives before being approved for administration.^33^

Despite the extensive research and formulation of thousands of anticancer nanomedicines with decent therapeutic efficiency in preclinical cancer models, only a few anticancer nanomedicines has been approved by regulatory agencies. This low success rate has been a matter of debate in the last decade. Testing of most of the nanomedicines is limited only to the preclinical stage, and only a few candidates that entered the early phases of clinical trials suffered high failure rates.

Three basic criteria are usually followed for the design of anticancer nanomedicines focusing on improving the anticancer efficacy and mitigating toxicity.^33^ The comparison of the tumor accumulation of nanomedicines is made only in tumors and not amongst the surrounding healthy tissues in most of the clinical studies. Therefore, the nanomedicine design based on tumor enhanced permeability and retention (EPR) or EPR heterogeneity may not be a suitable strategy for human cancer patients.^33^ Upon comparing Doxil with doxorubicin in ARKS lesions, it was found that the concentration of Doxil was 5.2 to 11.4 times greater than that of doxorubicin. However, given that ARKS arises from the spindle cells of the dermis tissue, it is not certain whether the increase in Doxil concentration in ARKS lesions is related to the EPR effect or preferred accumulation of Doxil in the epidermal and dermal tissues compared to the free doxorubicin.^33^ The superior efficacy of Doxil compared to doxorubicin may also be the reason for the preferred uptake of Doxil *versus* doxorubicin by spindle cells rather than the EPR effect in ARKS. Recently, Sindhwani *et al.* showed that the uptake of 97% of PEGylated gold nanoparticles (50 nm) by tumour cells is *via* endothelial cells rather than by the EPR effect. Therefore, we have to give serious thought and recheck the validity of the EPR criteria for the development of cancer nanomedicine, given that these studies reveal that EPR is certainly a simplification of complex clinical outcomes. In the same context, nanomedicines are intrinsically trapped in mouse xenograft tumors *via* the strong EPR effect, making their delivery highly efficient, but these enhancements are unlikely to be reflected in human cancers.^33^ Despite the differences in mouse xenograft cancer models and human cancer models, a wide array of nanomedicine research is being continuously conducted employing mouse xenograft cancer models.^33^ It is a matter of great concern that the enhanced delivery efficiency/efficacy of many nanodelivery systems in mouse xenograft cancer models may be an “artifact” of the animal model, which may be the principle reason for their poor clinical translation in human cancer patients.^33^ The efficiency of nanomedicines *versus* free drugs for human cancers can be well studied employing transgenic mouse spontaneous cancer models given that they closely resemble human cancers. Unfortunately, there is a lack of available spontaneous cancer models for different types of cancers. Also, the long systemic circulation should not be a universal nanomedicine design criterion. In the case of nanomedicines, long circulation times are helpful in elevating their tumor accumulation compared to the free drugs in preclinical xenograft cancer models owing to their strong EPR effect, but this may not be the case for better anticancer efficacy in human cancer patients.

The oversimplified expectations for nanomedicines for their clinical usage in cancer therapy should be discouraged, and thorough investigation of the designed nanomedicines should be performed. Multiple clinical failures have already been reported for the nanomedicines synthesized following simplified criteria of tumor EPR and long systemic circulation. Therefore, collaborative work is all that is needed at present. The application of nanomedicine research and fundamental basic research of nanotechnology must be balanced. Conversely, new materials should be employed to design novel nanostructures, which may not have any scope of being employed for human cancer therapies, but can be used to investigate fundamental theories of nanotechnology.

## 9. Conclusion

Despite the tremendous advancements made in cancer diagnosis and therapies, to date, no effective treatment has been found. All anti-cancer drugs used and available to treat cancer have potential side effects. NPs, owing to their maximum efficiency, specificity, and low toxicity, have received significant attention in the area of biomedicine; however, it depends on the type of cancer. Higher biocompatibility, lower aggregation rate, maximum clearance, and lower toxicity are among the most important factors to consider for the green synthesis of metal NPs (Ag, Au, Zn/ZnO, Cu/CuO), and research into their mechanism of action, cellular response, and *in vitro* therapeutic potential for various NP properties is still in its infancy. However, nanotechnology will be effectively utilized for the detection of transformed cells *via in vivo* imaging or ex vivo analysis in the early stage. This will allow the choice of the right combination of active ingredients based on accurate biological information about tumors, allowing for the targeted elimination of early cancer lesions with no side effects.

## Conflicts of interest

There are no conflicts to declare.
